# A replication study separates polymorphisms behind migraine with and without depression

**DOI:** 10.1371/journal.pone.0261477

**Published:** 2021-12-31

**Authors:** Peter Petschner, Daniel Baksa, Gabor Hullam, Dora Torok, Andras Millinghoffer, J. F. William Deakin, Gyorgy Bagdy, Gabriella Juhasz

**Affiliations:** 1 Bioinformatics Center, Institute for Chemical Research, Kyoto University, Gokasho, Uji, Kyoto, Japan; 2 Department of Pharmacodynamics, Faculty of Pharmacy, Semmelweis University, Budapest, Hungary; 3 MTA-SE Neuropsychopharmacology and Neurochemistry Research Group, Hungarian Academy of Sciences, Semmelweis University, Budapest, Hungary; 4 SE-NAP2 Genetic Brain Imaging Migraine Research Group, Hungarian Brain Research Program, Semmelweis University, Budapest, Hungary; 5 Department of Measurement and Information Systems, Budapest University of Technology and Economics, Budapest, Hungary; 6 NAP-2-SE New Antidepressant Target Research Group, Semmelweis University, Budapest, Hungary; 7 Neuroscience and Psychiatry Unit, Division of Neuroscience and Experimental Psychology, The University of Manchester and Manchester Academic Health Sciences Centre, Manchester, United Kingdom; Illumina Inc, UNITED STATES

## Abstract

The largest migraine genome-wide association study identified 38 candidate loci. In this study we assessed whether these results replicate on a gene level in our European cohort and whether effects are altered by lifetime depression. We tested SNPs of the loci and their vicinity with or without interaction with depression in regression models. Advanced analysis methods such as Bayesian relevance analysis and a neural network based classifier were used to confirm findings. Main effects were found for rs2455107 of *PRDM16* (OR = 1.304, p = 0.007) and five intergenic polymorphisms in 1p31.1 region: two of them showed risk effect (OR = 1.277, p = 0.003 for both rs11209657 and rs6686879), while the other three variants were protective factors (OR = 0.4956, p = 0.006 for both rs12090642 and rs72948266; OR = 0.4756, p = 0.005 for rs77864828). Additionally, 26 polymorphisms within *ADGRL2*, 2 in *REST*, 1 in *HPSE2* and 33 mostly intergenic SNPs from 1p31.1 showed interaction effects. Among clumped results representing these significant regions, only rs11163394 of *ADGRL2* showed a protective effect (OR = 0.607, p = 0.002), all other variants were risk factors (rs1043215 of *REST* with the strongest effect: OR = 6.596, p = 0.003). Bayesian relevance analysis confirmed the relevance of intergenic rs6660757 and rs12128399 (p31.1), rs1043215 (*REST*), rs1889974 (*HPSE2*) and rs11163394 (*ADGRL2*) from depression interaction results, and the moderate relevance of rs77864828 and rs2455107 of *PRDM16* from main effect analysis. Both main and interaction effect SNPs could enhance predictive power with the neural network based classifier. In summary, we replicated p31.1, *PRDM16*, *REST*, *HPSE2* and *ADGRL2* genes with classic genetic and advanced analysis methods. While the p31.1 region and *PRDM16* are worthy of further investigations in migraine in general, *REST*, *HPSE2* and *ADGRL2* may be prime candidates behind migraine pathophysiology in patients with comorbid depression.

## Introduction

Migraine is a serious neurological disorder characterized by recurrent headache, typically with unilateral pulsating pain aggravated by physical activity, nausea, photo- and/or phonophobia. Worldwide on average 11% of adults are affected, with women three times more often [1]. Migraineurs suffer severe interference with daily activities due to the disease that also manifests in high direct (e.g. healthcare service utilization) and indirect (including reduced productivity) costs [2–4]. Estimated annual expenses of migraine in Europe are around €27 billion [5] and the disease is responsible for 25 million lost school- and working days in the UK alone [1]. All these data motivate investigations of the pathomechanisms of the disease, which are still not fully understood.

The pathophysiology of common migraine involves multiple factors including genetic elements. Genetic heritability of the disease is around 40–50% [6] and several hypothesis-driven candidate gene studies reported associations, e.g. with polymorphisms of *HTR1A*, *HTR1B*, *HTR2A*, *MAOA*, *SLC6A4*, *COMT*, and *CNR1* genes, though their etiological roles are not certain [7–10]. Since migraine is polygenic in nature, genome-wide association studies (GWAS) that investigate polymorphisms associated with the disease along the entire genome, can point to novel candidates for further testing. A recent, seminal GWAS meta-analysis identified 38 loci associated with migraine [11]. After initial identification of such variants replication studies are necessary to verify and refine findings.

In addition to genetic risk, comorbid conditions may also play a role in the pathophysiology of migraine including psychiatric disorders, like depression [6, 12–15]. A recent study on 117,392 subjects from the UK Biobank cohort further supported a comorbid relationship between depression and migraine [16]. Depression also has a polygenic background and genetic association and twin studies suggest shared genetic risk factors between migraine and depression [17–20]. Based on GWAS results the genetic correlation (r_G_) between migraine and depression is between 0.25–0.32 [18, 21], however, migraine with and without comorbid depression might also develop on partially different genetic background [6] and a causal relationship is also possible [17]. Investigating monozygotic twins discordant for depression showed that only the twin suffering from depression has increased risk for migraine, indicating similar conclusions [17].

All these findings indicate that migraine has complex relationships involving several factors. Standard methods, however, are often inadequate to explore complex relationships such as nonlinear interactions. Advanced analysis tools utilizing machine learning methods may reveal additional aspects of variables and relationships. One such aspect is their relevance with respect to the disease. More precisely, which variables are strongly relevant, i.e. belong to the minimal Markov blanket of the target variable, which is migraine in this case. These variables represent the sufficient set of variables to uniquely define the value of the target variable in a graph representing dependencies [22]. We applied a method to determine strong relevance, the so called Bayesian network based Bayesian multilevel analysis of relevance, or Bayesian relevance analysis in short. This method was recently applied to analyze relevance of comorbidities, environmental factors and genetic variants [16, 23, 24].

The other important property of a variable is its predictive power. That is, how accurately a given variable predicts the target variable. Predictive power can be measured by using the variables as features in a classifier, such as a neural network, whose prediction accuracy is then assessed. Based on the above, both methods can complement traditional methods, such as linear or logistic regressions.

In light of the above, the aim of the present study was threefold: 1) to replicate the results of the largest migraine GWAS [11] by conducting a candidate gene study involving all polymorphisms within the 10.000 base pair (bp) vicinity of proposed genes/loci based on our independent samples from Budapest and Manchester using logistic regression models; 2) to investigate whether polymorphisms within the already proposed relevant genes/loci show interactions with lifetime depression with regression models; 3) to validate the identified single nucleotide polymorphisms (SNPs) with two machine learning algorithms, a Bayesian relevance analysis and a neural network based classifier to confirm the relevance, and the predictive power of the identified factors, respectively.

In summary, analyses presented in this paper validate polymorphisms in p31.1 region of chromosome 1, *PRDM16*, *ADGRL2*, *REST* and *HPSE2* genes in migraine. Furthermore, results allowed the separation of variants that play a role in migraine accompanied by depression, like *ADGRL2*, *REST* and *HPSE2*, from those that play a general role in migraine, like *PRDM16*. Finally, we also show, how different machine learning methods can be applied to analyze and evaluate effects of genetic polymorphisms interacting with non-genetic factors.

## Materials and methods

To achieve our objectives, first we conducted standard genetic association analyses of polymorphisms within the genes/loci identified by Gormley et al. [11] and their 10.000 base pair (bp) vicinity in two European population genetic samples (from Budapest and Manchester) and their combination. Subsequently, we performed interaction analyses with lifetime depression using the same samples. We used a less stringent significance criterion (p<0.05 in all three samples, for a justification see below), however we also applied two machine learning algorithms, a Bayesian relevance analysis method and a neural network classifier to confirm findings.

### Subjects

Around 2700 participants (aged between 18–60) were recruited through advertisements and general practices from Greater Manchester, United Kingdom and Budapest, Hungary. Ethics Committees in both cities approved the study (Scientific and Research Ethics Committee of the Medical Research Council, Budapest, Hungary, ad.225/KO/2005.; ad.323-60/2005-1018EKU and ad.226/KO/2005.; ad.323-61/2005-1018 EKU; North Manchester Local Research Ethics Committee, Manchester, UK REC reference number: 05/Q1406/26), and all our procedures were in accordance with the Declaration of Helsinki. Written informed consent was provided by all participants. We included subjects of European white origin (based on self-reported data) who provided DNA sample. Our total subject number was n = 1815 (in Budapest: n = 839; in Manchester: n = 975) after screening for available data on sex, age, ethnicity, migraine and lifetime depression status. For exact subject numbers used in each analysis, see S3–S8 Tables. Recruitment strategies and response rates were specified earlier [25].

### Phenotype

English and Hungarian versions of brief standard questionnaires were used in the study. Our validated background questionnaire [26] collected data about sex, age, ethnicity, sociodemographic status, personal and family psychiatric history. Information about lifetime depression (DEPR) also originated from this questionnaire. Lifetime depression was defined as a positive answer to reported lifetime depression in the personal psychiatric history of the background questionnaire. Furthermore, this was validated in a smaller subset of the original sample plus an independent sample by face-to-face diagnostic interviews as described previously [26].

The ID-Migraine questionnaire, a validated migraine-screening tool [27] was used to measure migraine. This questionnaire consists of 3 items measuring main symptoms of migraine in the last 3 months: photophobia, nausea and disability. Migraine (ID_MIGR) was defined as 2 or 3 YES answers to the questions about symptoms, and was validated earlier [27].

### Genotyping, quality control and imputation

A genetic saliva sample kit was used to collect DNA data–extracted from buccal mucosa cells [25]. Genotyping was implemented using Illumina’s CoreExom PsychChip yielding 573,141 variants with genomic position according to the Genome Reference Consortium Human 37 build (GRCh37/hg19). Details of quality control and imputation were published earlier [28] and were based on an established protocol [29]. Briefly, after restricting the measured variants to biallelic and autosomal polymorphisms, imputation was made using SHAPEIT (https://mathgen.stats.ox.ac.uk/genetics_software/shapeit/shapeit.html) and IMPUTE2 (http://mathgen.stats.ox.ac.uk/impute/impute_v2.html) softwares from the reference data. Quality control (QC) was performed for the different populations (Budapest, Manchester and total sample) separately and started with exclusion of multiallelic variants and variants with an ‘info’ and ‘certainty’ score of less than 0.5 and 0.7, respectively. Afterwards, the following thresholds were used for filtering: a minor allele frequency (MAF) of 0.01; iteratively 0.1, 0.05, and 0.01 missingness; a Hardy-Weinberg equilibrium test p-value ≥ 1x10^-5^; 0.2 of the squared correlation coefficient (R^2^) value for LD pruning; and an identical-by-descent (π^) value ≤ 0.1875. Principal components were also calculated to control for genetic heterogeneity. Individuals with uncertain gender (discrepancy between chromosome composition and questionnaire data), or outliers by heterozygosity were also excluded. SNPs were then filtered for the candidate genes/loci reported by Gormley et al. [11] and their 10,000 bp vicinity yielding 36579 candidate polymorphisms in 1815 individuals as a final dataset for subsequent analysis. Further details about quality control steps can be seen in S1 Appendix.

### Statistical analyses

#### Plink analysis

We used PLINK v1.07 analysis program (https://zzz.bwh.harvard.edu/plink/), the standard in genetic testing, to calculate Hardy-Weinberg equilibrium for individual SNPs (not on genome-wide level), and to run logistic regression analysis. SNPs yielding a Hardy-Weinberg p-value of less than 0.05 were excluded. In all regression analyses two-tailed tests were used.

ID_MIGR was our categorical outcome variable. The following covariates were added to every regression analysis: age, sex and the calculated first 10 principal components of the whole genetic dataset to control for population substructure (i.e. hidden subgroups as defined by their genetic compositions). First, we tested the main effects of the selected single nucleotide polymorphisms on migraine. After main effects analysis DEPR was added as an interacting variable to test SNP x DEPR interaction on migraine, representing the biological phenomenon that a SNP acts on migraine differently in those individuals who reported lifetime depression than in those who did not. We performed our analyses in our subsamples (Budapest and Manchester separately) and in the total sample (see S1 and S2 Tables for population descriptors).

In case of multiple genetic association tests, Bonferroni correction is often used to control multiple hypothesis testing [30–32]. In our study, however, we applied a more permissive significance criterion. The reasons were the followings: 1) Bonferroni correction is often criticized for its stringency on the field of genomics [33, 34] 2) most corrections assume independent tests, however, this is not the case in our analysis due to substantial linkage between polymorphisms (e.g. 1p31.1 region is a large haploblock (see Gormley et al. [11], supplementary materials)); 3) in contrast to exploratory, hypothesis-free GWASs, we wanted to confirm and extend robust findings proven by Gormley et al. [11], which allows for less stringent thresholds [35]; 4) recent modelling demonstrated that application of more permissive p-value thresholds could enhance the detection of true positive signals, which is more important in our replication study [36]; 5) it is well-known that both common migraine and depression are polygenic disorders with very small expected effect sizes for individual SNPs.

Therefore, we used permissive thresholds for p-values and SNPs were considered statistically significant according to the following criteria: 1) significant effect in both subsamples with p<0.05 and 2) replication of these results in the total sample with the same effect allele and effect direction, p<0.05. For comparison, if we only applied the criterion of a 0.05 significance in the total sample, altogether 1286 polymorphisms were significant. By restricting these SNPs to those showing a significant effect in both subsamples and also in the total sample we obtained only six (see Results). Note, however, that none of the SNPs remained significant using Bonferroni correction either in the main or interaction effect analyses (see also Limitations).

We also tested, whether any of the polymorphisms that were found significant with respect to migraine in main effect or interaction analyses, have an effect on lifetime depression. Logistic regression analysis was performed with the same covariates as for migraine, in the total sample. The significance threshold was the same as above.

For genotype and phenotype frequencies see S1 and S2 Tables. Standard methods like logistic/linear regression, however, are often inadequate to explore complex relationships such as nonlinear interactions. Advanced analysis tools utilizing machine learning methods may enable a more detailed analysis of relationships, especially in a multivariate case. Previously, we applied Bayesian relevance analysis in order to create a Bayesian direct multimorbidity map of depression and its comorbidities, investigating the direct and non-direct relationships of depression [16]; to characterize the relevance of relationships between depression and several environmental and lifestyle factors [23].

Another aspect of variables in addition to their relevance is their predictive power, which measures how accurately do individual or multiple variables in a model predict a target variable. In the present scenario this can be stated as: how accurately do genetic variables (SNPs) and the presence (or absence) of depression (as a comorbid disease) predict the migraine status of patients. For this purpose, we utilize a neural network classifier which is a universal approximator, capable of learning multivariate nonlinear relationships given adequate sample size. The application of such methods for the analysis of genetic associations is not as frequent as the presence of complex relationships. One of the goals of this study is to show that a detailed analysis requires additional tools besides the standard methods.

#### Bayesian relevance analysis

As a post-hoc test, we applied a Bayesian systems-based method called Bayesian relevance analysis [22], which relies on Bayesian model averaging [37]. The method considers possible variable dependency models fitting the data using a Markov chain Monte Carlo method to perform a random walk in the space of possible dependency models that are directed acyclic graphs [38], and assesses the probability of models and model properties [39]. The number of ‘burn-in’ steps were 1,000,000 followed by 2,000,000 additional steps starting from a randomly initialized structure, using 4 chains. The structure prior was set to uniform, and a Cooper-Herskovits prior (of the Bayesian Dirichlet prior family) was utilized as the parameter prior. Possible directed acyclic graph structures were limited such that each node could have at most 5 incoming edges (parents).

Variables that are directly connected to a selected target variable or those that form an interaction pattern with the target variable (please, note, this is not the same interaction, like the DEPR x SNP interaction parameter used above) in the majority of possible dependency models, can be considered strongly relevant [40].

We have to note that the Bayesian relevance analysis method tests all the SNPs jointly in a multivariate model and automatically corrects for multiple hypothesis testing, however, the method cannot correct automatically for a strong correlation of SNPs. Therefore, we used PLINK’s built-in clumping method to select important variants from relevant blocks in high LD. For clumping, the only relevant criterion was that from each block with an R^2^ higher than 0.6 only one SNP can be selected (for clumping results see S9 Table). Please, note, that throughout the manuscript relevance values represent relevance of the given variant with respect to migraine.

For additional information on Bayesian relevance analysis and strong relevance, see S2 Appendix.

#### Analysis of predictive power

We also utilized a neural network based classifier to assess the predictive power of relevant SNPs as a multivariate model. We created a neural network in the Tensorflow framework [41] which consisted of a fully connected input layer and a hidden layer using the most commonly used rectified linear unit (relu) activation function, and an output layer. Based on multiple experiments with various architectures, we found that more than 10 neurons per layer did not improve classification results. Therefore, we opted to use 10 neurons in both the input and hidden layers. Weights and biases were randomly initialized according to the uniform distribution. Since the target variable was binary, we utilized binary-cross entropy as a loss function. The ADAM method [42] was used as an optimizer with a batch size of 20 samples, and 50 as the number of learning epochs.

Predictive power was determined according to a weighted accuracy score involving sensitivity and specificity measures taking into account the case-control ratio. All measures were computed using a cross-validation framework of k = 10 partitions and n = 10 repetitions, i.e. the data was split into k = 10 partitions and for each classifier learning phase 9 partitions were used as training data and 1 partition as testing data, cycling through all possible combinations. This process starting with a partitioning was repeated n = 10 times. In other words, the average of each measure computed n x k (i.e. 100) times was used for the final evaluation. The same clumping method was used to filter SNPs prior to predictive power analysis as in the case of relevance analysis.

### Functional prediction

Functional characterization of the significant SNPs was performed with Functional Prediction tool, FuncPred (https://snpinfo.niehs.nih.gov/snpinfo/snpfunc.html) and with SNPnexus (https://www.snp-nexus.org/v4/). SNPnexus provided an opportunity to get a more detailed description of functionally relevant SNPs.

## Results

### Logistic regression results for main effect analysis

Among the 36579 polymorphisms only 6 showed significant (p<0.05) main effects for migraine that replicated in both subsamples (Budapest, Manchester) and in the total sample. From the six polymorphisms 5 (rs11209657, rs6686879, rs77864828, rs12090642, rs72948266) were located in the intergenic regions on the short arm of the first chromosome (1p31.1). Among them, based on the odds ratios (ORs) of the individual polymorphisms, 3 (rs77864828, rs12090642, rs72948266) represented protective factors (OR<1) and 2 (rs11209657, rs6686879) could be considered as risk factors for migraine (OR>1). The only polymorphism that could be associated with a gene was rs2455107 in the intronic region of *PRDM16* showing an OR higher than 1. For detailed results, see Table 1 and S3–S5 Tables. None of the above mentioned polymorphisms showed main effects with respect to lifetime depression.

**Table 1:** Significant genetic polymorphisms in main effects logistic regression analysis for migraine in the subsamples (Budapest, Manchester) and the total sample.

Variant name	Function prediction based on UCSC	Chromosome	Reference allele	Effect Allele	Associated gene / localization	Results of logistic regression models in main effect analyses					
						Budapest		Manchester		Total sample	
						p-value	OR	p-value	OR	p-value	OR
rs2455107	intron variant	1	A	C	*PRDM16*	0.0442	1.384	0.0463	1.288	0.0072	1.304
rs11209657	intergenic	1	G	A	chr1.p31.1	0.0477	1.298	0.0342	1.255	0.003	1.277
rs6686879	intergenic	1	G	A	chr1.p31.1	0.0477	1.298	0.0342	1.255	0.003	1.277
rs77864828	intergenic	1	C	T	chr1.p31.1	0.0295	0.3546	0.0478	0.526	0.0048	0.4756
rs12090642	intergenic	1	T	C	chr1.p31.1	0.0499	0.4215	0.0394	0.5128	0.0064	0.4956
rs72948266	intergenic	1	A	G	chr1.p31.1	0.0499	0.4215	0.0394	0.5128	0.0064	0.4956

**Table 1** shows significant polymorphisms, their respective functions based on the UCSC Genome Browser, chromosome localization, reference and effect allele, their associated gene or more exact genomic region, and the p-values and odds ratios (ORs) from the main effects logistic regression with age, sex, and 10 principal components as covariates. Note, that only one polymorphism could be associated with a gene, namely, *PRDM16* and all other variants are intergenic from a large LD block (p31.1) on the first chromosome. From the 38 investigated loci only these replicated according to our criteria.

### Logistic regression results for interaction effect analysis

51 polymorphisms showed a replicated (p<0.05 in both subsamples and total sample) effect in interaction with lifetime depression for migraine, from which 26 could be associated with *ADGRL2* (also known as *LPHN2*) on chromosome 1, with rs7412827 being a downstream gene variant, while the others were intron variants. Only 2 of them (rs11163394, rs3790895) could be considered protective. We also identified 22 intergenic risk polymorphisms on chromosome 1 in interaction with lifetime depression. One variant (rs1043215) in the 3’ untranslated region and another downstream variant (rs143167654) of *REST* (also known as *NRSF*) showed surprisingly high ORs for migraine in the interaction analyses [total sample OR: 6.596 (95% CI: 1.90–22.86), Manchester OR: 5.769 (95%: 1.07–31.04), Budapest OR: 10.5 (95% CI: 1.37–80.38) for both SNPs]. Rs1889974 of *HPSE2*, also showed significant effects. Clumped results representing the regions were collected in Table 2, for full results see S6–S8 Tables. None of the polymorphisms showed main effects for lifetime depression.

**Table 2:** Clumped results representing significant regions showing interaction with lifetime depression for migraine by logistic regression analysis.

Variant name	Function prediction based on UCSC	Chromosome	Reference allele	Effect allele	Associated gene / localization	Results of logistic regression models in interaction effect analyses					
						Budapest		Manchester		Total sample	
						p-value	OR	p-value	OR	p-value	OR
rs11163394	intron variant	1	G	A	*ADGRL2*	0.0491	0.5598	0.0015	0.4946	0.002	0.6079
rs6598982	intergenic	1	T	C	chr1.p31.1	0.0194	1.998	0.0024	2	0.0002	1.837
rs12128399	intergenic	1	G	T	chr1.p31.1	0.0116	2.268	0.0045	2.159	0.0004	1.923
rs12129408	intergenic	1	A	G	chr1.p31.1	0.0313	1.899	0.0274	1.67	0.02093	1.462
rs6660757	intergenic	1	A	C	chr1.p31.1	0.019	1.919	0.01	1.767	0.0001841	1.833
rs1043215	3’ UTR variant	4	G	A	*REST*	0.0235	10.5	0.0412	5.769	0.002939	6.596
rs1889974	intron variant	10	G	A	*HPSE2*	0.019	1.985	0.0265	1.647	0.002424	1.635

**Table 2** shows significant polymorphisms, their respective functions based on UCSC Genome Browser, chromosome localization, reference and effect allele, their associated gene or more exact genomic region, and the p-values and odds ratios (ORs) in interaction with lifetime depression for migraine using logistic regression with age, sex, and 10 principal components as covariates.

### Bayesian relevance analysis

To determine which of the identified polymorphisms are the most relevant for migraine we also ran a Bayesian relevance analysis investigating multivariate dependency structures on our total sample including sex, age, and a population descriptor (Budapest or Manchester). Significant SNPs from main effect analyses were checked for linkage and only the most representative SNPs were included in the Bayesian analysis (rs2455107, rs77864828, and rs11209657). Results for representative SNPs with main effects are presented in Table 3.

**Table 3:** Posterior probability of relevance with respect to migraine for SNPs with main effects.

Variant name	Relevance	Chromosome	Associated gene
rs77864828	0.421	1	intergenic
rs2455107	0.191	1	*PRDM16*
rs11209657	0.138	1	intergenic

**Table 3** shows posterior probabilities of main effect SNPs using a Bayesian relevance analysis. A higher posterior probability indicates higher relevance with respect to migraine and thus, serves as a post-hoc test for the already significant polymorphisms identified by regression models. Interestingly, the intergenic polymorphism rs77864828 shows a larger posterior probability, than the only gene-associated polymorphism rs2455107, *PRDM16*. Note that this method investigates possible multivariate models, i.e. it tests the SNPs jointly.

To distinguish between SNPs that showed significant effects in an interaction model, we performed Bayesian relevance analysis separately in depressed and non-depressed subjects on a clumped SNP set and calculated differences between relevance scores (i.e. posterior probabilities) (see Table 4). Four out of these polymorphisms showed at least a magnitude larger probability of relevance in depressed subjects than in non-depressed subjects and with the exception of rs11163394 all had an OR larger than 1 in regression models. The largest difference in relevance was found in the case of intergenic rs12128399 from the 1p31.1 region with low probability of relevance in non-depressed and high probability of relevance in depressed subjects on migraine. Rs12128399 was followed by rs6660757 in the p31.1 region, rs1889974 in *HPSE2*, rs1043215 in *REST* and rs11163394 in *ADGRL2* sorted by the relevance difference between depressed and non-depressed individuals.

**Table 4:** Posterior probability of relevance of SNPs with respect to migraine in interaction with lifetime depression.

Variant name	Relevance in non-depressed subjects	Relevance in depressed subjects	Difference in relevance between depressed and non-depressed subjects	Chromosome	Associated gene
rs12128399	0.025	0.929	0.904	1	Intergenic
rs6660757	0.210	0.997	0.787	1	Intergenic
rs1889974	0.047	0.704	0.657	10	*HPSE2*
rs1043215	0.290	0.907	0.617	4	*REST*
rs11163394	0.014	0.311	0.298	1	*ADGRL2*
rs6598982	0.013	0.059	0.047	1	Intergenic
rs12129408	0.0056	0.0062	0.0006	1	Intergenic

In **Table 4**, a higher posterior probability value indicates higher relevance with respect to migraine, shown separately for non-depressed and depressed subjects. A difference between relevance values of a SNP indicates that the SNP plays different roles in non-depressed and depressed subjects and, thus, confirms gene-disease interaction. With the exception of the intergenic rs12129408 where the difference is negligible, all of the polymorphisms show larger relevance in depressed individuals, suggesting that these polymorphisms are more likely to contribute to migraine in depressed subjects. Please, note that the model tests all SNPs at the same time. The low performance of rs12129408 is a result of multivariant effects discussed in the Limitations section.

### Predictive power analysis

We also investigated the predictive performance of three multivariate models related to either the main effects or the interactions of SNPs with respect to migraine (see Table 5). All models were compared to a baseline model (M0) consisting of age, sex and population. In this analysis, the population variable was selected instead of the 10 principal components for two reasons: 1) previously, we found that the first principal component is congruous with the population variable, and the addition of further principal components to the analysis does not change significantly the lambda-value of genomic inflation factor [43]; 2) this way, the results of the two machine learning algorithms are more comparable since the Bayesian relevance analysis can only handle categorical variables. The first model (M1) contained M0 and the SNPs with main effects with respect to migraine, the second model (M2) M0 and lifetime depression, and the third model (M3) those SNPs that were found relevant with respect to migraine in interaction with lifetime depression and can be considered as representative SNPs of their respective locality.

**Table 5:** Predictive power of multivariate models compared to a baseline containing age, sex and population variables.

Models		Accuracy	
		Score	Difference
M0	Age, sex, population	0.565	n/a
M1	Age, sex, population, rs2455107, rs11209657, rs77864828	0.664	17.56%
M2	Age, sex, population, lifetime depression	0.709	25.45%
M3	Age, sex, population, lifetime depression, rs11163394, rs6598982, rs12128399, rs12129408, rs6660757, rs1043215, rs1889974	0.767	35.76%

**Table 5** shows the predictive score calculated by a neural network based classifier and the relative difference from the base model using only age, sex and population to predict migraine. The predictive score is a weighted average of sensitivity and specificity measures computed using the total sample. All scores are compared to the score of the baseline model M0. In the M1 model, the significant main effect polymorphisms could enhance predictive power, nevertheless, the addition of the lifetime depression variable without any genetic variants (M2) achieved better performance. The further addition of the interaction polymorphisms to M2 (M3) yielded somewhat better results, showing that lifetime depression in itself is one of the best predictors of migraine, and probably reflecting the large effect size difference between lifetime depression and genetic variants.

Main effect SNPs could enhance predictive power by 17.56% when compared to the M0 model. Inclusion of lifetime depression enhanced the predictive power by 25.45% when compared to M0. Inclusion of the representative interaction SNPs in addition to lifetime depression, slightly improved predictions, resulting in a better score by 35.76% than that of M0 (and a 10.31% better score if compared to M2).

To confirm results of the predictive power analysis, we have investigated logistic regression models with the same terms used in models M0-M3 described in the predictive power analysis section; migraine served as the target variable. Specifically, M0: sex, age, population; M1: M0 + rs2455107, rs11209657, rs77864828; M2: M0 + DEPR; M3: M2 + rs12129408, rs6598982, rs11163394, rs12128399, rs1889974, rs1043215, rs6660757. Note that in case of M3 the interaction terms (formed between a SNP and depression) are modelled explicitly, e.g. DEPR: rs12129408.

Results are shown in S13 Table. In the case of M1, all 3 SNPs showing a main effect can be considered as significant terms of the logistic regression model. Furthermore, the depression descriptor variable in model M2 is highly significant (p-value: 1.39E-12), which further confirms that depression can be considered a relevant factor in the etiology of migraine. Model M3 contains the main effects and interactions terms of SNP found significant in interaction with depression. Among the 7 interaction terms only 3 are significant (DEPR:rs6660757, p-value: 6.75E-05; DEPR:rs1043215, p-value: 0.00275; rs1889974, p-value: 0.0096).

Due to the nature of the multivariate model, it is possible that given the effect of the most significant terms, other terms might appear insignificant. Therefore, we investigated each SNP individually in the sense that we formed variations of the M3 model such that only a single SNP (main effect and interaction with depression) was admitted to the model besides age, sex, population and depression. S14 Table shows the significance of interaction terms of such models. Results indicate that in each case the interaction term is significant to some extent even for those SNPs whose interaction term was not significant in the complex M3 model.

In addition, we compared the performance of the models using the Akaike information criterion (AIC). S15 Table shows the AIC score for each of the models. AIC is an estimator of prediction error, a lower value is considered better, furthermore it includes a penalty term for model complexity. Results confirm the outcome of predictive power analysis. M0 has the highest score (AIC: 2033.7) outperformed by M1 (AIC:2017.5) containing additional SNPs with main effects with respect to migraine. Model M2 containing the depression descriptor (AIC: 1984.9) is even better than M1 due to the significant effect of depression and the decreased complexity with respect to M1 (i.e. there are fewer variables in the model). Finally, M3 has to lowest score (AIC: 1954.3), indicating that the interaction of depression and the selected SNPs may play an important role in predicting migraine susceptibility.

### Functional prediction results

Functional prediction results can be seen in S10–S12 Tables. Among the significant polymorphisms of the main effect analysis, several showed indicative scores for functional effect. For example, rs6686879 showed high score based on gene annotation, while rs12090642 could have an effect as a transcription start site or as an eQTL variant (both SNPs are situated in the 1p31.1 region). Among the hits of interaction analysis, all variants showed scores suggesting functional effect, especially rs1043215 from *REST* gene.

## Discussion

Our analyses confirmed main effects of *PRDM16* and 1p31.1 SNPs on migraine in two independent European cohorts. Furthermore, this study also provides evidence that SNPs of the previously identified migraine risk genes *REST*, *ADGRL2*, *HPSE2* and 1p31.1 show lifetime depression-dependent associations with the disease. The neural network-based classifier proved the predictive value of both main and interaction effect SNPs on migraine, while Bayesian relevance analysis showed large relevance difference of the interaction SNPs between depressed and non-depressed migraineurs.

### Main effects

In 2016 Gormley and colleagues identified 38 credible sets of SNPs that may have a genetic impact on migraine [11]. To replicate the findings, we extracted 36579 polymorphisms (from them 25161 belonged to 1p31.1, a single, huge LD-block according to Gormley and colleagues [11]) that were in the vicinity of these loci. Our main effect analysis for migraine demonstrated only 6 nominally significant SNPs, and among them only one could be associated with a gene, the intron region of *PRDM16*: rs2455107. This polymorphism showed previously no associations, whatsoever, with migraine or any other diseases. At the same time, different variants of *PRDM16* associated with migraine in several earlier studies [11].

Rs2651899 of *PRDM16* showed associations in the Women’s Genome Health Study (WGHS) with migraine compared to non-migraineurs and also with migraine compared to non-migraine type headache [44]. A replication study on a Chinese population supported a role for rs2651899 as a risk factor in migraine without aura regardless of gender [45] and its role was also demonstrated in two North Indian, a Chinese Han, a Spanish, a Swedish, and a Pakistani population [46–51]. A recent meta-analysis also demonstrated an association between rs2651899 and migraine risk [52]. Risk allele carriers at rs2651899 also associated with larger response to serotonin 1B/1D receptor agonist antimigraine medications [53]. In contrast, Gormley et al. [11] suggested that rs10218452 and rs12135062 of *PRDM16* are responsible for main effects with respect to migraine.

Yet, we know little about the gene itself. *PRDM16* is implicated in brown adipose tissue differentiation that is a major contributor to thermoregulation and is capable for heat production [54, 55] (also according to the Kyoto Encyclopedia of Genes and Genomes (KEGG): https://www.kegg.jp/kegg-bin/search_pathway_text?map=&keyword=PRDM16&mode=1&viewImage=true). A case study reported association between body temperature and headache intensity in migraineurs with aura [56] and a hypothesis by Horvath suggests a causal role for altered thermoregulation in migraine [57]. From another perspective, *PRDM16* was shown to be necessary for adult neurogenesis (also see the AmiGO 2 database (Gene Ontology): http://amigo.geneontology.org/amigo/gene_product/UniProtKB:Q9HAZ2) and ependymal cell differentiation in the adult mouse brain after deletion of *PRDM16* with the Nestin-Cre method. In the latter study, the few remaining ciliated ependymal cells were abnormal and could not secure the proper flow of the cerebrospinal fluid (CSF) [58]. In chronic migraine, concentrations of several biomarkers are altered in CSF, e.g. glutamate or calcitonin-gene related peptide to name a few [59] that may connect altered ependymal cell function with the disease.

However, these links remain strongly hypothetic. Nonetheless, previous and current results indicate that polymorphisms of *PRDM16* may be consistent and relevant candidates for migraine susceptibility. The redundancy of the polymorphisms on the gene level, or allelic heterogeneity, suggests that altered gene functions or expression may be more important than individual variants within *PRDM16*.

#### Main effects—Bayesian relevance analysis and analysis of predictive power

Our post-hoc Bayesian relevance analysis showed the following order of relevance: 1) rs77864828 of 1p31.1 region; 2) rs2455107 of *PRDM16* and 3) rs11209657 of 1p31.1 region. Furthermore, inclusion of these 3 representative SNPs in the neural network classifier enhanced predictive power by 17.56% which is large considering that individual genetic variants have usually very small effects and the inclusion of lifetime depression could enhance predictive power within the same magnitude (25.45%). Gormley et al. [11] identified the 1p31.1 region as a risk locus and we can confirm these findings. Recent studies show that intronic and intergenic regions are responsible for harboring vast numbers of non-coding RNAs modulating gene transcription [60, 61]. These results point out that the fine modulation of transcription might be responsible for common migraine instead of large impact mutations.

In summary, main effect analyses including regression models and neural network based predictive power calculations indicated that rs2455107 of *PRDM16* and intergenic rs11209657, rs77864828 of 1p31.1 are potential candidate polymorphisms behind migraine. Other loci proposed by Gormley et al. [11] could not be replicated in our sample.

### Interaction effects

Interestingly, none of the polymorphisms that were significant in interaction effect analysis with lifetime depression showed significance in main effect results. Previous studies already suggested a substantial difference between migraine with and without depression [6, 17]. A twin study demonstrated that migraine and anxious depression may be in a causal relationship, though the direction of such causality remained undetermined [17]. The authors implied that migraine with and without depression can be regarded as different phenotypes and refuted the idea of a shared etiology [17]. In further support, according to polygenic risk scores, migraine with depression showed higher resemblance to depression than to migraine [6]. Our study adds further evidence for a substantial difference since there were candidate SNPs elevating the risk of migraine with comorbid lifetime depression that were not involved in mediating migraine in general. These results implicate that migraine with and without lifetime depression may develop on different genetic background, and that large GWASs might find SNPs relevant in specific subgroups of the disorder. In the future, these SNPs can be used for personalized risk/phenotype prediction (like in [62, 63]). The regression analysis identified 51 SNPs from three genes. We discuss them in the context of the results from the two machine learning methods.

#### Interaction effects—Bayesian relevance analysis and analysis of predictive power

Post-hoc analysis showed that among the identified SNPs the two most relevant ones were rs12128399 and rs6660757 of p31.1, both are intergenic and their exact role is at present unknown.

The third polymorphism, rs1889974, based on relevance difference between depressed and non-depressed migraineurs, is located in the intron of *HPSE2*, encoding the heparanase-2 (Hpa2) enzyme [64]. Hpa2 was identified as a novel member of the heparanase family [64]. Heparanases are endoglycosidases cleaving the heparan sulfate (HS), which leads to the remodelling of the extracelullar matrix [64], and is connected to tumor metastasis, angiogenesis and inflammation, but Hpa2 does not exhibit this HS-degrading activity and is not a subject of proteolytic processing [65]. Its wild type (Hpa2c) has an inhibitory effect on heparanase enzymatic activity, likely through Hpa2c’s high affinity to HS and heparin [65]. Our result with *HPSE2* might support the vascular theory of migraine. A prothrombotic tendency has been suggested in migraine pathogenesis by the connection between migraine and cardio- and cerebrovascular ischemic events, although related studies show conflicting data [66–68] and the literature of anticoagulants’ efficacy in migraine patients also shows mixed results [66]. Depression has been connected to cardiovascular diseases too [69, 70] and the ‘vascular depression’ hypothesis also links cerebrovascular disease to geriatric depressive syndromes [71, 72]. In addition, *HPSE2* associated with the depression-related trait, neuroticism [73]. These results may represent a lead connecting the gene with migraine and depression.

The fourth largest relevance difference was found in case of rs1043215, which is located within the *REST* gene, an important regulator of gene expression with the ability to bind RE1 (also known as NRSF) in the promoter region of more than 2000 genes [74–76]. In non-neuronal cells *REST* is responsible for the suppression of neuron specific gene expression during embryogenesis [77]. In adult neuronal cells *REST* is expressed at low levels and is induced by different types of neuronal activation [78]. Among the regulated genes in neuronal cell lines were synapsin, synaptophysin, Crh, *BDNF*, and the serotonin 1A receptor [74, 79, 80], which are highly relevant for depression and antidepressant response [81]. In fact, aberrant *REST* transcriptional regulation was shown in patients with major depressive disorder in current depressive state, but not in remission [79]. While it remained so far uninvestigated with respect to migraine directly, *REST* was also involved in neuropathic pain symptoms [82]. Furthermore, decreased REST levels associated with increased neuronal excitability and cortical activity that may be directly related to elevated pain sensations [83]. The two identified polymorphisms of *REST* showed large ORs for migraine. The relevant SNP in the Bayesian analysis, rs1043215 in the 3’ untranslated region was uninvestigated previously. However, our functional prediction analysis showed that this SNP is functional with great probability, and it was detected as a highly conserved SNP suggesting an evolutionary importance for this variant. The above discussed empirical evidences and the present study delineate *REST* polymorphisms, especially rs1043215, as possible candidates for further studies in migraine with depression.

The next SNP according to relevance difference, rs11163394, belongs to an intronic region of *ADGRL2* from 1p31.1 encoding latrophilin 2, an adhesion G-protein coupled receptor [84]. Latrophilin receptors are involved in the maintenance of synapses and *ADGRL2* in particular is involved in the intracellular release of Ca-ions [85]. While the latrophilin 1 and 3 receptors were proposed as contributors to the regulation of mood [85, 86], *ADGRL2* remains hitherto less investigated. The reason was probably the relatively low expression of *ADGRL2* compared to other latrophilins, within the brain [87], however, it cannot be excluded that *ADGRL2* is also involved in the regulation of mood. Elevated excitability of tissues via calcium-channelopathies were already proposed in migraine [88]. In certain cases, familial hemiplegic migraine is caused by mutations in the calcium voltage-gated channel subunit alpha1 A (CACNA1A), and it was also proposed that physiologic cation concentrations may be altered in other forms of the disease [88, 89]. Our study suggests that via variation in the *ADGRL2* gene, intracellular calcium levels might be elevated, resulting in a hyperexcitability similar to that seen in the familial form of the disease. While it may be premature to draw conclusions about the role of *ADGRL2* in migraine with accompanying depression, the 26 different polymorphisms associated with *ADGRL2* show that the gene might represent an important novel target of migraine research.

The last two variants based on relevance difference were rs6598982 and rs12129408 from the 1p31.1 region. Our functional prediction analysis showed high scores for rs6598982 in some categories suggesting high probability for being an eQTL, a trait-associated and inherited disease-associated variant. Furthermore, it has to be noted that non-coding *LINC01360* and *RN7SKP19* pseudogene in the region associated with depression and anxiety disorder in GWASs [90–93]. From the perspective of the predictive power, inclusion of these interacting SNPs in the model slightly improved its prediction value compared to adding only lifetime depression (10.31% gain).

In summary, interaction analysis indicated that intergenic rs12128399, rs6660757 and variants in *HPSE2*, *REST* and from the 1p31.1 region (including *ADGRL2*) represent lifetime depression-dependent genetic factors for migraine.

### Limitations

Our study has to be interpreted in light of the following limitations. First, our sample is small compared to the original study. The lack of large statistical power may explain why we couldn’t replicate many loci from the Gormley study. To remain rigorous, our significance criterion was to replicate results in both subsamples and the total sample with nominal significance instead of the Bonferroni correction used in large genetic studies. We applied two machine learning methods leading to further validation and characterization of results: the Bayesian relevance analysis validated relevance of interaction results, while the predictive power analysis confirmed both main effect and interaction polymorphisms. The lack of Bonferroni correction remains a major limitation of the study. We already discussed why we choose more permissive p-value thresholds at the relevant section of Methods.

Second, we also have to note that the two subsamples differ in some characteristics (see S1 and S2 Tables), but exactly for this very reason, we believe that those SNPs that were able to replicate are indeed important.

Third, we measured migraine and lifetime depression with questionnaires. The migraine questionnaire was already validated against a clinical sample as demonstrated by Lipton and colleagues [27, 94]. The latter is also true for the depression questionnaire, which was validated by us, as indicated in the methods section [95].

Fourth, the polymorphism rs12129408 showed risk OR (> 1) in the PLINK analysis, but the difference between relevance in depressed and non-depressed individuals was negligible. This can be explained by the different aims of the methods. The PLINK analysis assesses effect sizes and based on these estimations calculates significance. At the same time, Bayesian relevance analysis addresses whether a given polymorphism has a direct effect on the outcome variable, but does not state whether this effect is protective or not.

Fifth, the minor allele frequency of the *REST* gene polymorphism was low in our sample that might explain the large ORs seen in the analyses (see S2 Table). Therefore, these results should be interpreted with caution.

Finally, predictive power analysis could confirm the effect of main effect SNPs, and also the effect of interaction SNPs to some extent, while Bayesian relevance analysis validated interaction effects only. It has to be noted, that these two methods answer different questions about the roles of these SNPs (predictive power and direct effects, respectively) and, therefore, it is not surprising that different results were obtained.

## Conclusions

The elaborate analysis of 38 risk loci for migraine already proposed by the GWAS of Gormley et al. [11] could identify only 57 nominally significant variants from altogether four genes/regions (*PRDM16*, *REST*, *ADGRL2*, *HPSE2*). Main effect analysis indicated that intergenic polymorphism rs77864828, and with smaller relevance, intron variant rs2455107 in *PRDM16* represent genetic factors for common migraine. In contrast, intergenic rs6660757 and rs12128399, rs1889974 in *HPSE2*, rs11163394 in *ADGRL2* and rs1043215 of *REST* may only serve as biomarkers in migraine accompanied by lifetime depression. In addition, while most of the above genes could be connected to known migraine pathophysiology, the 1p31.1 region harboring significant intergenic SNPs also assumes a role for non-coding RNAs behind common migraine.

The relative scarcity of significantly validated variants emphasize the need of validation even for large GWAS results especially through the inclusion of interaction factors. It also reveals that even significantly replicated genetic variants may be involved in different disease subtypes, in this particular case, migraine without and with accompanying lifetime depression.

## Supporting information

S1 Fig Genomic location of the significant SNPs in *PRDM16* gene. (PDF)

S2 Fig Genomic location of the significant SNPs from 1.p31.1 region. (PDF)

S3 Fig Genomic location of the significant SNPs near *REST* gene. (PDF)

S4 Fig Genomic location of the significant SNPs in *HPSE2* gene. (PDF)

S1 Table Summary statistics. (PDF)

S2 Table Minor allele frequencies. (PDF)

S3 Table Results for main effect term in Budapest subsample. (PDF)

S4 Table Results for main effect term in Manchester subsample. (PDF)

S5 Table Results for main effect term in total sample. (PDF)

S6 Table Results for interaction term in Budapest subsample. (PDF)

S7 Table Results for interaction term in Manchester subsample. (PDF)

S8 Table Results for interaction term in total sample. (PDF)

S9 Table Formatted results of the clumping procedure as performed in Plink on the total sample. (PDF)

S10 Table Results of the functional characterization with GWAVA non-coding scoring algorithm. (PDF)

S11 Table Results of the functional characterization with DeepSEA non-coding scoring algorithm. (PDF)

S12 Table Results of the functional characterization with FunSeq2 non-coding scoring algorithms. (PDF)

S13 Table Multivariate logistic regression models corresponding to models M0-M3 used for assessing predictive power. (PDF)

S14 Table Significance of interaction terms in variations of logistic regression model M3 each containing only one relevant SNP. (PDF)

S15 Table Evaluation of logistic regression models M0-M3 using Akaike information criterion (AIC). (PDF)

S1 Appendix Quality control (QC) steps. (PDF)

S2 Appendix Additional information on methods. (PDF)
