# Supplementary material for: A replication study separates polymorphisms behind migraine with and without depression
Source: PLoS One. 2021 Dec 31;16(12):e0261477. doi: 10.1371/journal.pone.0261477 (PMC8719675; doi:10.1371/journal.pone.0261477)
Supplement: S1 Fig — (PDF) [file pone.0261477.s001.pdf]

S1 Fig.: Genomic location of the significant SNPs in *PRDM16* gene

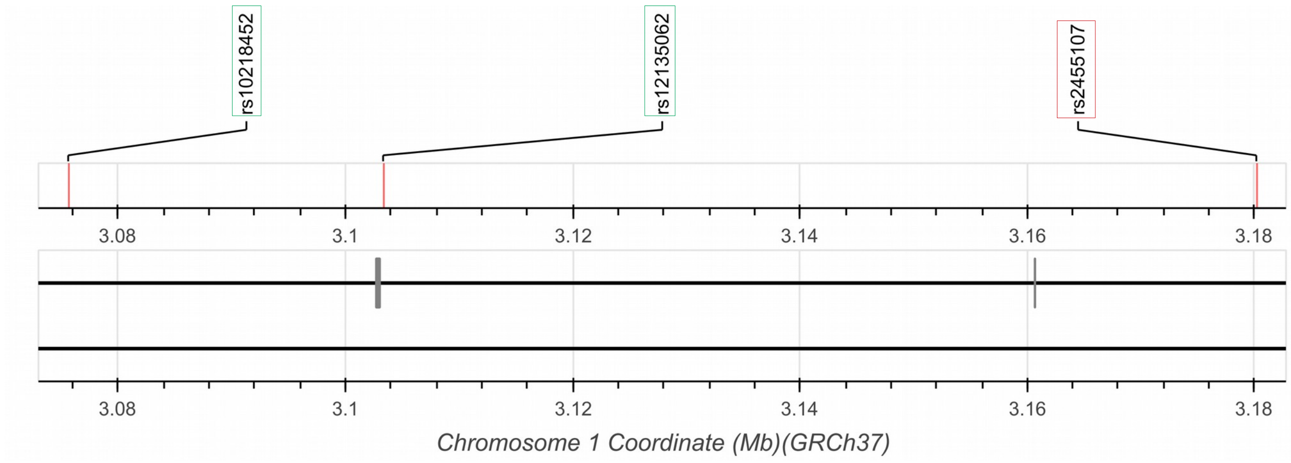

S1 Fig.: shows comparison of genomic location of the significant hits from *PRDM16* gene, from our study (rs2455107, red colour) and from the study of Gormley et al. (rs10218452, rs12135062, green colour). Despite their proximity to each other, these SNPs are independent (LD<0.2).
