# Supplementary material for: A replication study separates polymorphisms behind migraine with and without depression
Source: PLoS One. 2021 Dec 31;16(12):e0261477. doi: 10.1371/journal.pone.0261477 (PMC8719675; doi:10.1371/journal.pone.0261477)
Supplement: S2 Fig — (PDF) [file pone.0261477.s002.pdf]

S2 Fig.: Genomic location of the significant SNPs from 1.p31.1 region

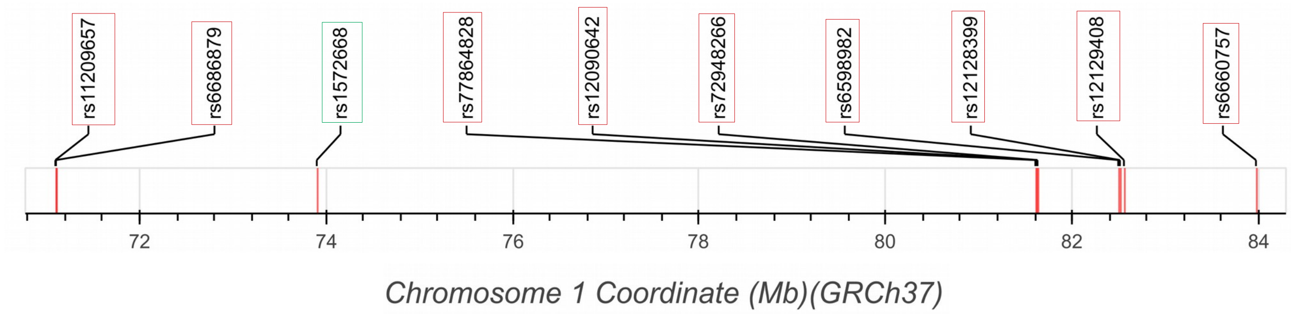

S2 Fig.: shows comparison of genomic location of the significant hits from 1.p31.1 region, from our study (rs11209657, rs6686879, rs77864828, rs12090642, rs72948266, rs6598982, rs12128399, rs12129408, rs6660757, red colour) and the lead SNP from the study of Gormley et al. (rs1572668, green colour). The lead SNP from the study of Gormley et al. and our hits are independent (LD<0.2).
