# Supplementary material for: A replication study separates polymorphisms behind migraine with and without depression
Source: PLoS One. 2021 Dec 31;16(12):e0261477. doi: 10.1371/journal.pone.0261477 (PMC8719675; doi:10.1371/journal.pone.0261477)
Supplement: S3 Fig — (PDF) [file pone.0261477.s003.pdf]

S3 Fig.: Genomic location of the significant SNPs near *REST* gene

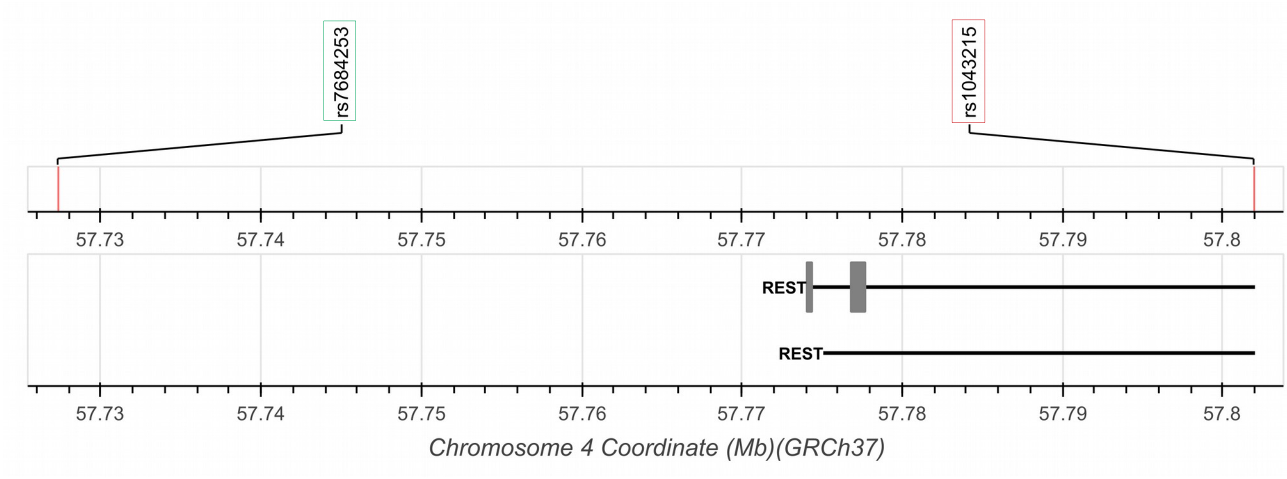

S3 Fig. shows comparison of genomic location of the significant hits near *REST* gene, from our study (rs1043215, red colour) and the lead SNP from the study of Gormley et al. (rs7684253 green colour). The two SNPs are independent of each other (LD<0.2).
