# Supplementary material for: A replication study separates polymorphisms behind migraine with and without depression
Source: PLoS One. 2021 Dec 31;16(12):e0261477. doi: 10.1371/journal.pone.0261477 (PMC8719675; doi:10.1371/journal.pone.0261477)
Supplement: S1 Table — (PDF) [file pone.0261477.s005.pdf]

S1 Table: Summary statistics

Sample	Budapest subsample	Manchester subsample	Total sample
**Age**			
Min	18	18	18
Max	60	60	60
Mean	30.83	34.05	32.62
Median	28	34	31
**Gender**			
Males	271 (32.30%)	263 (26.97%)	534 (29.42%)
Females	568 (67.70%)	712 (73.03%)	1281 (70.58%)
**Depression**			
No	665 (79.26%)	429 (44.00%)	1095 (60.33%)
Yes	174 (20.74%)	546 (56.00%)	720 (39.67%)
**Migraine**			
No	661 (78.78%)	667 (68.41%)	1328 (73.17%)
Yes	178 (21.22%)	308 (31.59%)	487 (26.83%)

S1 Table shows demographic and phenotype distributions in Budapest, Manchester subsamples and in total sample.
