# Supplementary material for: A replication study separates polymorphisms behind migraine with and without depression
Source: PLoS One. 2021 Dec 31;16(12):e0261477. doi: 10.1371/journal.pone.0261477 (PMC8719675; doi:10.1371/journal.pone.0261477)
Supplement: S2 Table — (PDF) [file pone.0261477.s006.pdf]

S2 Table: Minor allele frequencies

Main effect hits	Minor allele	Budapest subsample	Manchester subsample	Total sample	Reference population (EUR)
rs2455107	C	0.1784	0.1800	0.1791	0.1839
rs11209657	A	0.3258	0.3002	0.3125	0.339
rs6686879	A	0.3258	0.3002	0.3125	0.339
rs77864828	T	0.03571	0.03049	0.0329	0.0338
rs12090642	C	0.03627	0.03096	0.0329	0.0358
rs72948266	G	0.03627	0.03096	0.0329	0.0358
**Interaction hits**	**Minor allele**	**Budapest subsample**	**Manchester subsample**	**Total sample**	**Reference population (EUR)**
rs11163394	A	0.3711	0.4105	0.3919	0.4205
rs6598982	C	0.4570	0.4294	0.4424	0.5477
rs12128399	T	0.2923	0.2515	0.2705	0.2803
rs12129408	G	0.4346	0.3945	0.4131	0.5984
rs6660757	C	0.4411	0.4133	0.4259	0.6074
rs1043215	A	0.0210	0.02645	0.0239	0.0298
rs1889974	A	0.4126	0.3971	0.4038	0.6233

S2 Table shows minor allele frequencies of the significant SNPs from the main effect and interaction analyses in case of Budapest, Manchester subsamples and total sample, and in the reference sample (European population from 1000Genome Project).
