# Supplementary material for: A replication study separates polymorphisms behind migraine with and without depression
Source: PLoS One. 2021 Dec 31;16(12):e0261477. doi: 10.1371/journal.pone.0261477 (PMC8719675; doi:10.1371/journal.pone.0261477)
Supplement: S3 Table — (PDF) [file pone.0261477.s007.pdf]

S3 Table: Results for main effect term in Budapest subsample.

CHR	SNP	Effect allele	TEST	NMISS	OR	SE	L95	U95	STAT	P
1	rs2455107	C	ADD	784	1.384	0.1614	1.009	1.899	2.013	0.04417
1	rs11209657	A	ADD	784	1.298	0.1315	1.003	1.679	1.98	0.04771
1	rs6686879	A	ADD	784	1.298	0.1315	1.003	1.679	1.98	0.04771
1	rs77864828	T	ADD	783	0.3546	0.4763	0.1394	0.9018	-2.177	0.02948
1	rs12090642	C	ADD	784	0.4215	0.4405	0.1778	0.9995	-1.961	0.04988
1	rs72948266	G	ADD	784	0.4215	0.4405	0.1778	0.9995	-1.961	0.04988

S3 Table shows significant SNPs of main effect analysis in Budapest subsample. Logistic regression was performed with Plink v1.07, where migraine (ID_MIGR) acted as dependent variable, age, sex and the first 10 principal components were added as covariates.

Abbreviations:

CHR: chromosome code, SNP: single nucleotide polymorphism (rsID), Effect allele: the allele responsible for the effect, TEST: type of the model during statistical analyses, ADD: additive, NMISS: number of observations, OR: odds ratio, SE: standard error, L95: lower confidence interval, U95: upper confidence interval, STAT: t-statistic, p: asymptotic p-value for t-statistic.
