# Supplementary material for: A replication study separates polymorphisms behind migraine with and without depression
Source: PLoS One. 2021 Dec 31;16(12):e0261477. doi: 10.1371/journal.pone.0261477 (PMC8719675; doi:10.1371/journal.pone.0261477)
Supplement: S4 Table — (PDF) [file pone.0261477.s008.pdf]

S4 Table: Results for main effect term in Manchester subsample

CHR	SNP	Effect allele	TEST	NMISS	OR	SE	L95	U95	STAT	P
1	rs2455107	C	ADD	973	1.288	0.1271	1.004	1.653	1.992	0.04634
1	rs11209657	A	ADD	973	1.255	0.1071	1.017	1.548	2.118	0.03421
1	rs6686879	A	ADD	973	1.255	0.1071	1.017	1.548	2.118	0.03421
1	rs77864828	T	ADD	971	0.526	0.3247	0.2783	0.9938	-1.979	0.04781
1	rs12090642	C	ADD	972	0.5128	0.3241	0.2717	0.968	-2.06	0.03936
1	rs72948266	G	ADD	972	0.5128	0.3241	0.2717	0.968	-2.06	0.03936

S4 Table shows significant SNPs of main effect analysis in Manchester subsample. Logistic regression was performed with Plink v1.07, where migraine (ID_MIGR) acted as dependent variable, age, sex and the first 10 principal components were added as covariates.
Abbreviations:
CHR: chromosome code, SNP: single nucleotide polymorphism (rsID), Effect allele: the allele responsible for the effect, TEST: type of the model during statistical analyses, ADD: additive, NMISS: number of observations, OR: odds ratio, SE: standard error, L95: lower confidence interval, U95: upper confidence interval, STAT: t-statistic, p: asymptotic p-value for t-statistic.
