# Supplementary material for: A replication study separates polymorphisms behind migraine with and without depression
Source: PLoS One. 2021 Dec 31;16(12):e0261477. doi: 10.1371/journal.pone.0261477 (PMC8719675; doi:10.1371/journal.pone.0261477)
Supplement: S5 Table — (PDF) [file pone.0261477.s009.pdf]

S5 Table: Results for main effect term in total sample

CHR	SNP	Effect allele	TEST	NMISS	OR	SE	L95	U95	STAT	P
1	rs2455107	C	ADD	1757	1.304	0.09881	1.074	1.583	2.686	0.007233
1	rs11209657	A	ADD	1757	1.277	0.08244	1.087	1.501	2.968	0.003002
1	rs6686879	A	ADD	1757	1.277	0.08244	1.087	1.501	2.968	0.003002
1	rs77864828	T	ADD	1754	0.4756	0.2633	0.2838	0.7968	-2.822	0.004766
1	rs12090642	C	ADD	1756	0.4956	0.2575	0.2992	0.821	-2.726	0.006414
1	rs72948266	G	ADD	1756	0.4956	0.2575	0.2992	0.821	-2.726	0.006414

S5 Table shows significant SNPs of main effect analysis in total sample. Logistic regression was performed with Plink v1.07, where migraine (ID_MIGR) acted as dependent variable, age, sex and the first 10 principal components were added as covariates.
Abbreviations:
CHR: chromosome code, SNP: single nucleotide polymorphism (rsID), Effect allele: the allele responsible for the effect, TEST: type of the model during statistical analyses, ADD: additive, NMISS: number of observations, OR: odds ratio, SE: standard error, L95: lower confidence interval, U95: upper confidence interval, STAT: t-statistic, p: asymptotic p-value for t-statistic.
