# Supplementary material for: A replication study separates polymorphisms behind migraine with and without depression
Source: PLoS One. 2021 Dec 31;16(12):e0261477. doi: 10.1371/journal.pone.0261477 (PMC8719675; doi:10.1371/journal.pone.0261477)
Supplement: S6 Table — (PDF) [file pone.0261477.s010.pdf]

S6 Table: Results for interaction term in Budapest subsample

CHR	SNP	Effect allele	TEST	NMISS	OR	SE	L95	U95	STAT	P
1	rs284217	T	ADDxDEPR	784	2.481	0.337	1.282	4.803	2.696	0.007008
1	rs284216	G	ADDxDEPR	784	2.481	0.337	1.282	4.803	2.696	0.007008
1	rs284215	G	ADDxDEPR	784	2.481	0.337	1.282	4.803	2.696	0.007008
1	rs284213	C	ADDxDEPR	784	2.481	0.337	1.282	4.803	2.696	0.007008
1	rs284211	C	ADDxDEPR	784	2.481	0.337	1.282	4.803	2.696	0.007008
1	rs665458	G	ADDxDEPR	784	2.481	0.337	1.282	4.803	2.696	0.007008
1	rs284225	A	ADDxDEPR	784	2.481	0.337	1.282	4.803	2.696	0.007008
1	rs434619	T	ADDxDEPR	784	2.481	0.337	1.282	4.803	2.696	0.007008
1	rs412378	G	ADDxDEPR	784	2.481	0.337	1.282	4.803	2.696	0.007008
1	rs447267	G	ADDxDEPR	784	2.481	0.337	1.282	4.803	2.696	0.007008
1	rs651533	T	ADDxDEPR	782	2.542	0.3378	1.311	4.929	2.761	0.005758
1	rs284227	C	ADDxDEPR	782	2.542	0.3378	1.311	4.929	2.761	0.005758
1	rs284221	T	ADDxDEPR	784	2.585	0.3378	1.333	5.011	2.811	0.004931
1	rs284222	C	ADDxDEPR	784	2.585	0.3378	1.333	5.011	2.811	0.004931
1	rs284218	G	ADDxDEPR	780	2.492	0.3382	1.284	4.836	2.7	0.006931
1	rs284219	G	ADDxDEPR	779	2.458	0.3384	1.266	4.771	2.658	0.007863
1	rs11163394	A	ADDxDEPR	781	0.5598	0.2949	0.3141	0.9978	-1.968	0.04912
1	rs3790895	C	ADDxDEPR	784	0.5544	0.2933	0.312	0.9851	-2.011	0.04429
1	rs398254	A	ADDxDEPR	784	2.372	0.3257	1.253	4.491	2.652	0.008004
1	rs385367	G	ADDxDEPR	784	2.372	0.3257	1.253	4.491	2.652	0.008004
1	rs379975	T	ADDxDEPR	781	1.849	0.2919	1.044	3.277	2.107	0.03515
1	rs943366	C	ADDxDEPR	784	2.372	0.3257	1.253	4.491	2.652	0.008004
1	rs1327021	T	ADDxDEPR	784	2.372	0.3257	1.253	4.491	2.652	0.008004
1	rs12759788	G	ADDxDEPR	781	1.849	0.2919	1.044	3.277	2.107	0.03515
1	rs9438724	C	ADDxDEPR	776	2.397	0.3276	1.262	4.556	2.669	0.007606
1	rs7412827	A	ADDxDEPR	784	1.792	0.2869	1.021	3.145	2.034	0.04196
1	rs2038974	C	ADDxDEPR	780	2.165	0.3239	1.148	4.084	2.385	0.01708
1	rs12145656	G	ADDxDEPR	784	2.224	0.3247	1.177	4.202	2.461	0.01386
1	rs6598982	C	ADDxDEPR	780	1.998	0.2962	1.118	3.569	2.336	0.01947
1	rs12027404	G	ADDxDEPR	780	1.998	0.2962	1.118	3.569	2.336	0.01947
1	rs4262589	T	ADDxDEPR	783	1.957	0.2919	1.104	3.468	2.3	0.02142
1	rs4970660	A	ADDxDEPR	783	2.197	0.3235	1.166	4.143	2.434	0.01494
1	rs4970643	T	ADDxDEPR	784	1.966	0.2918	1.11	3.483	2.317	0.02048
1	rs4970661	T	ADDxDEPR	784	2.224	0.3247	1.177	4.202	2.461	0.01386
1	rs11163413	T	ADDxDEPR	784	1.966	0.2918	1.11	3.483	2.317	0.02048
1	rs12759645	A	ADDxDEPR	784	2.224	0.3247	1.177	4.202	2.461	0.01386
1	rs4400657	A	ADDxDEPR	784	2.227	0.3234	1.182	4.198	2.476	0.01329
1	rs4291539	C	ADDxDEPR	784	1.966	0.2918	1.11	3.483	2.317	0.02048
1	rs6690297	T	ADDxDEPR	784	2.227	0.3234	1.182	4.198	2.476	0.01329
1	rs4439384	T	ADDxDEPR	784	2.227	0.3234	1.182	4.198	2.476	0.01329
1	rs10782773	A	ADDxDEPR	784	1.966	0.2918	1.11	3.483	2.317	0.02048
1	rs11163414	T	ADDxDEPR	784	1.966	0.2918	1.11	3.483	2.317	0.02048
1	rs4970663	C	ADDxDEPR	783	2.211	0.3236	1.172	4.169	2.451	0.01423
1	rs4970644	C	ADDxDEPR	783	2.207	0.3249	1.167	4.172	2.436	0.01487
1	rs10874282	G	ADDxDEPR	783	1.966	0.2917	1.11	3.483	2.318	0.02048
1	rs12128399	T	ADDxDEPR	777	2.268	0.3244	1.201	4.284	2.525	0.01158
1	rs12129408	G	ADDxDEPR	784	1.899	0.298	1.059	3.407	2.153	0.03133
1	rs6660757	C	ADDxDEPR	784	1.919	0.2779	1.113	3.308	2.344	0.01906
4	rs1043215	A	ADDxDEPR	779	10.5	1.038	1.372	80.38	2.265	0.02352
4	rs143167654	A	ADDxDEPR	779	10.5	1.038	1.372	80.38	2.265	0.02352
6	rs10456623	A	ADDxDEPR	784	0.4467	0.4025	0.203	0.9832	-2.002	0.04528
10	rs1889974	A	ADDxDEPR	784	1.985	0.2914	1.121	3.514	2.352	0.01865

S6 Table shows significant SNPs of interaction analysis in Budapest subsample. Logistic regression was performed with Plink v1.07, where migraine (ID_MIGR) acted as dependent variable, age, sex and the first 10 principal components were added as covariates. Lifetime depression (DEPR) was added as an interacting variable to test SNP x DEPR interaction on migraine.

Abbreviations:

CHR: chromosome code, SNP: single nucleotide polymorphism (rsID), Effect allele: the allele responsible for the effect, TEST: type of the model during statistical analyses,

ADDxDEPR: additive model in interaction with lifetime depression, NMISS: number of observations, OR: odds ratio, SE: standard error, L95: lower confidence interval, U95: upper confidence interval, STAT: t-statistic, p: asymptotic p-value for t-statistic.
