# Supplementary material for: A replication study separates polymorphisms behind migraine with and without depression
Source: PLoS One. 2021 Dec 31;16(12):e0261477. doi: 10.1371/journal.pone.0261477 (PMC8719675; doi:10.1371/journal.pone.0261477)
Supplement: S7 Table — (PDF) [file pone.0261477.s011.pdf]

S7 Table: Results for interaction term in Manchester subsample

CHR	SNP	Effect allele	TEST	NMISS	OR	SE	L95	U95	STAT	P
1	rs284217	T	ADDxDEPR	970	1.811	0.2729	1.061	3.092	2.176	0.02953
1	rs284216	G	ADDxDEPR	970	1.811	0.2729	1.061	3.092	2.176	0.02953
1	rs284215	G	ADDxDEPR	970	1.811	0.2729	1.061	3.092	2.176	0.02953
1	rs284213	C	ADDxDEPR	970	1.811	0.2729	1.061	3.092	2.176	0.02953
1	rs284211	C	ADDxDEPR	970	1.811	0.2729	1.061	3.092	2.176	0.02953
1	rs665458	G	ADDxDEPR	970	1.811	0.2729	1.061	3.092	2.176	0.02953
1	rs284225	A	ADDxDEPR	970	1.811	0.2729	1.061	3.092	2.176	0.02953
1	rs434619	T	ADDxDEPR	969	1.799	0.2728	1.054	3.071	2.152	0.03137
1	rs412378	G	ADDxDEPR	969	1.799	0.2728	1.054	3.071	2.152	0.03137
1	rs447267	G	ADDxDEPR	970	1.809	0.2728	1.06	3.088	2.174	0.02971
1	rs651533	T	ADDxDEPR	967	1.798	0.2728	1.053	3.069	2.151	0.0315
1	rs284227	C	ADDxDEPR	969	1.799	0.2726	1.054	3.07	2.154	0.03123
1	rs284221	T	ADDxDEPR	971	1.782	0.2724	1.045	3.038	2.12	0.03399
1	rs284222	C	ADDxDEPR	971	1.782	0.2724	1.045	3.038	2.12	0.03399
1	rs284218	G	ADDxDEPR	969	1.817	0.2729	1.064	3.102	2.187	0.02872
1	rs284219	G	ADDxDEPR	969	1.817	0.2729	1.064	3.102	2.187	0.02872
1	rs11163394	A	ADDxDEPR	970	0.4946	0.2214	0.3205	0.7633	-3.18	0.001471
1	rs3790895	C	ADDxDEPR	973	0.5008	0.2208	0.3248	0.772	-3.132	0.001739
1	rs398254	A	ADDxDEPR	973	1.822	0.2725	1.068	3.108	2.201	0.02771
1	rs385367	G	ADDxDEPR	973	1.822	0.2725	1.068	3.108	2.201	0.02771
1	rs379975	T	ADDxDEPR	973	1.667	0.2265	1.069	2.598	2.255	0.02411
1	rs943366	C	ADDxDEPR	973	1.822	0.2725	1.068	3.108	2.201	0.02771
1	rs1327021	T	ADDxDEPR	973	1.822	0.2725	1.068	3.108	2.201	0.02771
1	rs12759788	G	ADDxDEPR	973	1.667	0.2265	1.069	2.598	2.255	0.02411
1	rs9438724	C	ADDxDEPR	965	1.862	0.2753	1.086	3.194	2.259	0.02387
1	rs7412827	A	ADDxDEPR	973	1.703	0.2212	1.104	2.628	2.407	0.01608
1	rs2038974	C	ADDxDEPR	964	2.142	0.2737	1.253	3.663	2.784	0.005374
1	rs12145656	G	ADDxDEPR	973	1.983	0.2697	1.169	3.365	2.539	0.01112
1	rs6598982	C	ADDxDEPR	971	2	0.2287	1.278	3.131	3.032	0.002433
1	rs12027404	G	ADDxDEPR	971	2	0.2287	1.278	3.131	3.032	0.002433
1	rs4262589	T	ADDxDEPR	973	1.814	0.2298	1.156	2.845	2.591	0.009577
1	rs4970660	A	ADDxDEPR	971	2.139	0.2708	1.258	3.636	2.808	0.004989
1	rs4970643	T	ADDxDEPR	973	1.814	0.2298	1.156	2.845	2.591	0.009577
1	rs4970661	T	ADDxDEPR	973	1.983	0.2697	1.169	3.365	2.539	0.01112
1	rs11163413	T	ADDxDEPR	973	1.814	0.2298	1.156	2.845	2.591	0.009577
1	rs12759645	A	ADDxDEPR	973	1.983	0.2697	1.169	3.365	2.539	0.01112
1	rs4400657	A	ADDxDEPR	973	2.11	0.2705	1.242	3.586	2.761	0.00577
1	rs4291539	C	ADDxDEPR	972	1.795	0.23	1.144	2.818	2.544	0.01095
1	rs6690297	T	ADDxDEPR	972	2.097	0.2706	1.234	3.563	2.736	0.006217
1	rs4439384	T	ADDxDEPR	972	2.097	0.2706	1.234	3.563	2.736	0.006217
1	rs10782773	A	ADDxDEPR	973	1.8	0.23	1.147	2.824	2.555	0.01063
1	rs11163414	T	ADDxDEPR	972	1.795	0.23	1.144	2.818	2.544	0.01095
1	rs4970663	C	ADDxDEPR	972	2.097	0.2706	1.234	3.563	2.736	0.006217
1	rs4970644	C	ADDxDEPR	972	1.97	0.2698	1.161	3.343	2.514	0.01195
1	rs10874282	G	ADDxDEPR	971	1.772	0.2302	1.129	2.783	2.485	0.01295
1	rs12128399	T	ADDxDEPR	967	2.159	0.2713	1.269	3.675	2.837	0.004556
1	rs12129408	G	ADDxDEPR	973	1.67	0.2323	1.059	2.633	2.206	0.02736
1	rs6660757	C	ADDxDEPR	973	1.767	0.2213	1.145	2.727	2.573	0.01008

S7 Table shows significant SNPs of interaction analysis in Manchester subsample.
Logistic regression was performed with Plink v1.07, where migraine (ID_MIGR) acted as dependent variable, age, sex and the first 10 principal components were added as covariates.
Lifetime depression (DEPR) was added as an interacting variable to test SNP x DEPR interaction on migraine.
Abbreviations:
CHR: chromosome code, SNP: single nucleotide polymorphism (rsID), Effect allele: the allele responsible for the effect, TEST: type of the model during statistical analyses, ADDxDEPR: additive model in interaction with lifetime depression, NMISS: number of observations, OR: odds ratio, SE: standard error, L95: lower confidence interval, U95: upper confidence interval, STAT: t-statistic, p: asymptotic p-value for t-statistic.
