# Supplementary material for: A replication study separates polymorphisms behind migraine with and without depression
Source: PLoS One. 2021 Dec 31;16(12):e0261477. doi: 10.1371/journal.pone.0261477 (PMC8719675; doi:10.1371/journal.pone.0261477)
Supplement: S8 Table — (PDF) [file pone.0261477.s012.pdf]

S8 Table: Results for interaction term in total sample

CHR	SNP	Effect allele	TEST	NMISS	OR	SE	L95	U95	STAT	P
1	rs284217	T	ADDxDEPR	1754	1.799	0.1869	1.247	2.595	3.141	0.001685
1	rs284216	G	ADDxDEPR	1754	1.799	0.1869	1.247	2.595	3.141	0.001685
1	rs284215	G	ADDxDEPR	1754	1.799	0.1869	1.247	2.595	3.141	0.001685
1	rs284213	C	ADDxDEPR	1754	1.799	0.1869	1.247	2.595	3.141	0.001685
1	rs284211	C	ADDxDEPR	1754	1.799	0.1869	1.247	2.595	3.141	0.001685
1	rs665458	G	ADDxDEPR	1754	1.799	0.1869	1.247	2.595	3.141	0.001685
1	rs284225	A	ADDxDEPR	1754	1.799	0.1869	1.247	2.595	3.141	0.001685
1	rs434619	T	ADDxDEPR	1753	1.795	0.1869	1.244	2.589	3.129	0.001753
1	rs412378	G	ADDxDEPR	1753	1.795	0.1869	1.244	2.589	3.129	0.001753
1	rs447267	G	ADDxDEPR	1754	1.802	0.1869	1.249	2.598	3.15	0.001633
1	rs651533	T	ADDxDEPR	1749	1.808	0.1871	1.253	2.609	3.165	0.001552
1	rs284227	C	ADDxDEPR	1751	1.812	0.187	1.256	2.615	3.179	0.001478
1	rs284221	T	ADDxDEPR	1755	1.81	0.1869	1.255	2.611	3.175	0.0015
1	rs284222	C	ADDxDEPR	1755	1.81	0.1869	1.255	2.611	3.175	0.0015
1	rs284218	G	ADDxDEPR	1749	1.82	0.1872	1.261	2.627	3.199	0.001378
1	rs284219	G	ADDxDEPR	1748	1.815	0.1872	1.257	2.619	3.183	0.001459
1	rs11163394	A	ADDxDEPR	1751	0.6079	0.1609	0.4436	0.8333	-3.094	0.001975
1	rs3790895	C	ADDxDEPR	1757	0.6107	0.1604	0.4459	0.8364	-3.073	0.002116
1	rs398254	A	ADDxDEPR	1757	1.812	0.1849	1.261	2.603	3.213	0.001315
1	rs385367	G	ADDxDEPR	1757	1.812	0.1849	1.261	2.603	3.213	0.001315
1	rs379975	T	ADDxDEPR	1754	1.603	0.1616	1.168	2.201	2.921	0.003485
1	rs943366	C	ADDxDEPR	1757	1.812	0.1849	1.261	2.603	3.213	0.001315
1	rs1327021	T	ADDxDEPR	1757	1.812	0.1849	1.261	2.603	3.213	0.001315
1	rs12759788	G	ADDxDEPR	1754	1.603	0.1616	1.168	2.201	2.921	0.003485
1	rs9438724	C	ADDxDEPR	1741	1.823	0.1866	1.265	2.628	3.219	0.001288
1	rs7412827	A	ADDxDEPR	1757	1.575	0.1587	1.154	2.15	2.863	0.004201
1	rs2038974	C	ADDxDEPR	1744	1.878	0.1839	1.31	2.693	3.427	0.0006109
1	rs12145656	G	ADDxDEPR	1757	1.821	0.1835	1.271	2.609	3.266	0.001093
1	rs6598982	C	ADDxDEPR	1751	1.837	0.1638	1.332	2.532	3.712	0.0002057
1	rs12027404	G	ADDxDEPR	1751	1.837	0.1638	1.332	2.532	3.712	0.0002057
1	rs4262589	T	ADDxDEPR	1756	1.683	0.1632	1.222	2.317	3.191	0.001419
1	rs4970660	A	ADDxDEPR	1754	1.89	0.183	1.32	2.705	3.477	0.0005064
1	rs4970643	T	ADDxDEPR	1757	1.688	0.1631	1.226	2.324	3.211	0.001324
1	rs4970661	T	ADDxDEPR	1757	1.821	0.1835	1.271	2.609	3.266	0.001093
1	rs11163413	T	ADDxDEPR	1757	1.688	0.1631	1.226	2.324	3.211	0.001324
1	rs12759645	A	ADDxDEPR	1757	1.821	0.1835	1.271	2.609	3.266	0.001093
1	rs4400657	A	ADDxDEPR	1757	1.888	0.1828	1.319	2.702	3.476	0.0005088
1	rs4291539	C	ADDxDEPR	1756	1.676	0.1632	1.217	2.308	3.163	0.001559
1	rs6690297	T	ADDxDEPR	1756	1.881	0.1829	1.315	2.692	3.456	0.0005489
1	rs4439384	T	ADDxDEPR	1756	1.881	0.1829	1.315	2.692	3.456	0.0005489
1	rs10782773	A	ADDxDEPR	1757	1.678	0.1632	1.218	2.31	3.17	0.001522
1	rs11163414	T	ADDxDEPR	1756	1.676	0.1632	1.217	2.308	3.163	0.001559
1	rs4970663	C	ADDxDEPR	1755	1.873	0.183	1.308	2.68	3.428	0.0006083
1	rs4970644	C	ADDxDEPR	1755	1.806	0.1837	1.26	2.588	3.217	0.001297
1	rs10874282	G	ADDxDEPR	1754	1.667	0.1632	1.211	2.296	3.131	0.001744
1	rs12128399	T	ADDxDEPR	1744	1.923	0.1835	1.342	2.755	3.562	0.0003681
1	rs12129408	G	ADDxDEPR	1757	1.462	0.1646	1.059	2.019	2.309	0.02093
1	rs6660757	C	ADDxDEPR	1757	1.833	0.1621	1.334	2.519	3.74	0.0001841
4	rs1043215	A	ADDxDEPR	1749	6.596	0.6343	1.903	22.86	2.974	0.002939
4	rs143167654	A	ADDxDEPR	1749	6.596	0.6343	1.903	22.86	2.974	0.002939
6	rs10456623	A	ADDxDEPR	1757	0.5125	0.2144	0.3366	0.7802	-3.117	0.001826
10	rs1889974	A	ADDxDEPR	1757	1.635	0.1622	1.19	2.247	3.033	0.002424

S8 Table shows significant SNPs of interaction analysis in the total sample. Logistic regression was performed with Plink v1.07, where migraine (ID_MIGR) acted as dependent variable, age, sex and the first 10 principal components were added as covariates. Lifetime depression (DEPR) was added as an interacting variable to test SNP x DEPR interaction on migraine.

Abbreviations:

CHR: chromosome code, SNP: single nucleotide polymorphism (rsID), Effect allele: the allele responsible for the effect, TEST: type of the model during statistical analyses, ADDxDEPR: additive model in interaction with lifetime depression, NMISS: number of observations, OR: odds ratio, SE: standard error, L95: lower confidence interval, U95: upper confidence interval, STAT: t-statistic, p: asymptotic p-value for t-statistic.
