# Supplementary material for: A replication study separates polymorphisms behind migraine with and without depression
Source: PLoS One. 2021 Dec 31;16(12):e0261477. doi: 10.1371/journal.pone.0261477 (PMC8719675; doi:10.1371/journal.pone.0261477)
Supplement: S9 Table — (PDF) [file pone.0261477.s013.pdf]

S9 Table: Formatted results of the clumping procedure as performed in Plink on the total sample

Clumped SNP	Status of Clumped SNP	SNP	BP	Distance from tag/non-tagSNP
rs398254	Tag	rs398254	82413673	Distance from tagSNP
		rs284217	82356110	57563
		rs284216	82356242	57431
		rs284215	82356897	56776
		rs284213	82357941	55732
		rs284211	82358699	54974
		rs665458	82360669	53004
		rs284225	82366667	47006
		rs434619	82373273	40400
		rs412378	82374494	39179
		rs447267	82375080	38593
		rs651533	82375561	38112
		rs284227	82379446	34227
		rs284221	82389853	23820
		rs284222	82390296	23377
		rs284218	82393466	20207
		rs284219	82395236	18437
		rs385367	82415199	-1526
		rs943366	82427833	-14160
		rs1327021	82428554	-14881
rs11163394	Non-Tag	rs11163394	82398752	Distance from non-tagSNP
		rs3790895	82401515	-2763
rs379975	Tag	rs379975	82419574	Distance from tagSNP
		rs12759788	82429259	-9685
		rs7412827	82462304	-42730
rs12128399	Tag	rs12128399	82512209	Distance from tagSNP
		rs9438724	82438148	74061
		rs2038974	82485561	26648
		rs12145656	82492195	20014
		rs4970660	82499606	12603
		rs4970661	82500277	11932
		rs12759645	82502547	9662
		rs4400657	82502635	9574
		rs6690297	82504289	7920
		rs4439384	82505010	7199
		rs4970663	82509065	3144
		rs4970644	82509390	2819
rs6598982	Tag	rs6598982	82494232	Distance from tagSNP
		rs12027404	82495835	-1603
		rs4262589	82498808	-4576
		rs4970643	82499691	-5459
		rs11163413	82501011	-6779
		rs4291539	82503816	-9584
		rs10782773	82508628	-14396
		rs11163414	82508794	-14562
		rs10874282	82510916	-16684
rs1043215	Tag	rs1043215	57801922	Distance from tagSNP
		rs143167654	57805470	-3548
rs12129408	Unique	rs12129408	82555368	-

S9 Table shows clumping results on the total sample. Clumping was performed in Plink software to select important variants from the relevant block with high LD. Thus, we selected one SNP from each block, with an R^2 higher than 0.6.
