# Supplementary material for: A replication study separates polymorphisms behind migraine with and without depression
Source: PLoS One. 2021 Dec 31;16(12):e0261477. doi: 10.1371/journal.pone.0261477 (PMC8719675; doi:10.1371/journal.pone.0261477)
Supplement: S10 Table — (PDF) [file pone.0261477.s014.pdf]

S10 Table: Results of the functional characterization with GWAVA non-coding scoring algorithm

Main effect analysis				
**rsID**	**Variant**	**Chr**	**Region Score**	**TSS Score**
rs2455107	C	1	0.25	0
rs11209657	A	1	0.21	0.17
rs6686879	A	1	0.47	0.22
rs77864828	T	1	0.33	0.17
rs12090642	C	1	0.33	0.35
rs72948266	G	1	0.41	0.11
**Interaction analysis**				
**rsID**	**Variant**	**Chr**	**Region Score**	**TSS Score**
rs11163394	A	1	0.3	0.2
rs6598982	C	1	0.44	0.35
rs12128399	T	1	0.28	0.21
rs12129408	G	1	0.38	0.21
rs6660757	C	1	0.26	0.09
rs1889974	A	10	0.25	0.14
rs1043215	A	4	0.57	0.7

S10 Table shows functional characterization of the significant SNPs, marked with rsID. The variant column contains the detected effect alleles. Region score, TSS Score are two different prediction scores calculated by GWAVA (Genome Wide Annotation of VAriants) algorithm. Region score is a prediction of function based of known summary gene region annotations. TSS (transcription start site) score is calculated from the prediction of the distance to the nearest annotated transcription start site. Both scores are in the range [0-1]. The higher the scores, the greater the probability to be functional. Rs1043215 from *REST* gene shows the highest scores.
