# Supplementary material for: A replication study separates polymorphisms behind migraine with and without depression
Source: PLoS One. 2021 Dec 31;16(12):e0261477. doi: 10.1371/journal.pone.0261477 (PMC8719675; doi:10.1371/journal.pone.0261477)
Supplement: S11 Table — (PDF) [file pone.0261477.s015.pdf]

S11 Table: Results of the functional characterization with DeepSEA non-coding scoring algorithm

Main effect analysis						
**rsID**	**Variant**	**Chr**	**Functional Significance Score**	**eQTL Probability**	**GWAS Probability**	**HGMD Probability**
rs2455107	C	1	0.092401	0.59537	0.66112	0.47859
rs11209657	A	1	0.32964	0.42481	0.39361	0.39712
rs6686879	A	1	0.076469	0.61808	0.55207	0.44198
rs77864828	T	1	0.047469	0.47635	0.75878	0.47807
rs12090642	C	1	0.21147	0.71241	0.59721	0.42149
rs72948266	G	1	0.29662	0.17231	0.29558	0.31362
**Interaction analysis**						
**rsID**	**Variant**	**Chr**	**Functional Significance Score**	**eQTL Probability**	**GWAS Probability**	**HGMD Probability**
rs11163394	A	1	0.17874	0.66169	0.54849	0.40643
rs6598982	C	1	0.017158	0.65867	0.77681	0.5179
rs12128399	T	1	0.11561	0.53342	0.6066	0.46427
rs12129408	G	1	0.42513	0.45158	0.49266	0.42023
rs6660757	C	1	0.099474	0.48543	0.32296	0.41632
rs1889974	A	10	0.28094	0.6014	0.676	0.44019
rs1043215	A	4	0.004014	0.84534	0.7519	0.48853

S11 Table shows functional characterization of the significant SNPs, marked with rsID. The variant column contains the detected effect alleles. Functional significance score predicts the significance of predicted chromatin effect and evolutionary conservation. It ranges from 0 to 1, lower scores indicate functional significance. eQTL Probability denotes the probability that the given variant is an eQTL variant.

GWAS probability predicts that the observed variant is a trait-associated (GWAS) variant. HGMD Probability denotes the probability that the observed variant is an inherited disease-associated variant, based on the data from Human Gene Mutation Database (HGMD). All three Probability score ranges from 0 to 1, the higher scores indicate higher probabilities.
