# Supplementary material for: A replication study separates polymorphisms behind migraine with and without depression
Source: PLoS One. 2021 Dec 31;16(12):e0261477. doi: 10.1371/journal.pone.0261477 (PMC8719675; doi:10.1371/journal.pone.0261477)
Supplement: S12 Table — (PDF) [file pone.0261477.s016.pdf]

**S12 Table**: Results of the functional characterization with FunSeq2 non-coding scoring algorithms

| **Main effect analysis** | | | |
|---|---|---|---|
| **rsID** | **Variant** | **Chr** | **Non-coding score** |
| rs2455107 | C | 1 | 0.201041 |
| rs11209657 | A | 1 | 0 |
| rs6686879 | A | 1 | 0.000693 |
| rs77864828 | T | 1 | 0 |
| rs12090642 | C | 1 | 0.155386 |
| rs72948266 | G | 1 | 0 |
| **Interaction analysis** | | | |
| **rsID** | **Variant** | **Chr** | **Non-coding score** |
| rs11163394 | A | 1 | 0.108736 |
| rs6598982 | C | 1 | 0 |
| rs12128399 | T | 1 | 2.8E-05 |
| rs12129408 | G | 1 | 0.155386 |
| rs6660757 | C | 1 | 0.155386 |
| rs1889974 | A | 10 | 0.011479 |
| rs1043215 | A | 4 | 1.610218 |

**S12 Table** shows functional characterization of the significant SNPs, marked with rsID. The variant column contains the detected effect alleles. FunSeq2 algorithm calculates non-coding score for a given variant. In general, it ranges from 0 to 1, the higher the scores, the greater the probability to be functional. In case of rs1043215, we can observe non-coding score more than 1. This can occur in case of highly conserved polymorphisms with GERP (Genomic Evolutionary Rate Profiling) score higher than 2. GERP score for rs1043215 is 4.78, which suggests the evolutionary importance of this SNP.
