# Supplementary material for: A replication study separates polymorphisms behind migraine with and without depression
Source: PLoS One. 2021 Dec 31;16(12):e0261477. doi: 10.1371/journal.pone.0261477 (PMC8719675; doi:10.1371/journal.pone.0261477)
Supplement: S13 Table — (PDF) [file pone.0261477.s017.pdf]

S13 Table: Multivariate logistic regression models corresponding to models M0-M3 used for assessing predictive power.

M0	Estimate	Std. Error	P-value	Significance
Sex	-0.97578	0.13743	1.25E-12	***
Age_range	-0.05624	0.0509	0.269	
Population	0.52702	0.11155	2.31E-06	***
**M1**	**Estimate**	**Std. Error**	**P-value**	**Significance**
Sex	-0.98451	0.13825	1.07E-12	***
Age_range	-0.06469	0.05119	0.20632	
Population	0.54428	0.11248	1.30E-06	***
rs2455107	0.25339	0.09766	0.00947	**
rs11209657	0.21611	0.08102	0.00764	**
rs77864828	-0.70382	0.25778	0.00633	**
**M2**	**Estimate**	**Std. Error**	**P-value**	**Significance**
Sex	-0.8849	0.13928	2.11E-10	***
Age_range	-0.08228	0.05184	0.1125	
Population	0.23796	0.12046	0.0482	*
DEPR	0.83743	0.1182	1.39E-12	***
**M3**	**Estimate**	**Std. Error**	**P-value**	**Significance**
Sex	-0.88672	0.141999	4.25E-10	***
Age_range	-0.07666	0.053105	0.14885	
Population	0.242625	0.123143	0.04881	*
DEPR	-0.26509	0.451179	0.55683	
rs11163394	0.164875	0.158029	0.2968	
rs6598982	0.006883	0.191241	0.97129	
rs12128399	-0.16085	0.23818	0.49945	
rs12129408	0.058471	0.182874	0.74917	
rs6660757	-0.16371	0.114017	0.15106	
rs1043215	-0.99786	0.531361	0.06039	
rs1889974	-0.09182	0.116554	0.4308	
DEPR:rs11163394	-0.19967	0.220468	0.36513	
DEPR:rs6598982	0.105878	0.266009	0.69061	
DEPR:rs12128399	0.537833	0.344776	0.11877	
DEPR:rs12129408	-0.1264	0.262834	0.63058	
DEPR:rs6660757	0.648159	0.162656	6.75E-05	***
DEPR:rs1043215	1.924554	0.642828	0.00275	**
DEPR:rs1889974	0.419805	0.162098	0.0096	**

Std.Error stands for standard error. (*) denotes moderately significant terms with p-value<0.05, (**) denotes significant terms with p-value<0.01, and (***) denotes highly significant terms with p-value<0.001.
