# Supplementary material for: A replication study separates polymorphisms behind migraine with and without depression
Source: PLoS One. 2021 Dec 31;16(12):e0261477. doi: 10.1371/journal.pone.0261477 (PMC8719675; doi:10.1371/journal.pone.0261477)
Supplement: S14 Table — (PDF) [file pone.0261477.s018.pdf]

S14 Table: Significance of interaction terms in variations of logistic regression model M3 each containing only one relevant SNP.

Interaction term	Estimate	Std. Error	P-value	Significance
DEPR:rs12129408	0.35503	0.1621	0.02851	*
DEPR:rs6598982	0.52066	0.15952	0.0011	**
DEPR:rs11163394	-0.41752	0.15807	0.00826	**
DEPR:rs12128399	0.63483	0.17978	0.000414	***
DEPR:rs1889974	0.44936	0.15885	0.00467	**
DEPR:rs1043215	1.9434	0.634	0.00217	**
DEPR:rs6660757	0.64372	0.15995	5.71E-05	***

Std.Error stands for standard error. (*) denotes moderately significant terms with p-value<0.05, (**) denotes significant terms with p-value<0.01, and (***) denotes highly significant terms with p-value<0.001.
