# Supplementary material for: A replication study separates polymorphisms behind migraine with and without depression
Source: PLoS One. 2021 Dec 31;16(12):e0261477. doi: 10.1371/journal.pone.0261477 (PMC8719675; doi:10.1371/journal.pone.0261477)
Supplement: S15 Table — (PDF) [file pone.0261477.s019.pdf]

S15 Table: Evaluation of logistic regression models M0-M3 using Akaike information criterion (AIC).

Model	Terms	AIC
M0	Sex, Age, Population	2033.7
M1	Sex, Age, Population, rs2455107, rs11209657, rs77864828	2017.5
M2	Sex, Age, Population, DEPR	1984.9
M3	Sex, Age, Population, DEPR, rs12129408, rs6598982, rs11163394, rs12128399, rs1889974, rs1043215, rs6660757	1954.3
