# Supplementary material for: A replication study separates polymorphisms behind migraine with and without depression
Source: PLoS One. 2021 Dec 31;16(12):e0261477. doi: 10.1371/journal.pone.0261477 (PMC8719675; doi:10.1371/journal.pone.0261477)
Supplement: S1 Appendix — (PDF) [file pone.0261477.s020.pdf]

S1 Appendix Quality control (QC) steps.

Our quality control (QC) procedure (published previously by Eszlari et al., 2019) included the following steps:

- restricting the measured variants to biallelic and autosomal polymorphisms;
- strand alignments of these polymorphisms were checked against the reference data of IMPUTE2 (https://mathgen.stats.ox.ac.uk/impute/1000GP_Phase3.html);
- imputation was made in two steps:
    1) SHAPEIT (https://mathgen.stats.ox.ac.uk/genetics_software/shapeit/shapeit.html) was used to determine haplotype information, then
    2) IMPUTE2 (http://mathgen.stats.ox.ac.uk/impute/impute_v2.html) was used to impute missing variants of reference data, yielding a total of 81,613,199 variants;
- exclusion of multiallelic and not single nucleotide variants and variants with an info and ceraintу of less than 0.5 and 0.7, respectively.

From this point, QC-steps were preformed separately for both subsamples (Manchester and Budapest) and the total sample. The used filtering tresholds for exclusion and dimensions of resulting datasets (sample count × number of variants) were the followings:

- a minor allele frequency (MAF) of 0.01 (Budapest: 850×7817868, Manchester: 1112×7842330, Total: 1962×7830425);
- iteratively 0.1, 0.05, and 0.01 missingness;
- a Hardy-Weinberg equilibrium test p-value ≥ 1x10-5.

After this step, the remaining SNPs were retained for the analysis, but further filtering were used for the samples with the following thresholds:

- 0.2 of of the squared correlation coefficient (R2) value for LD pruning;
- and an identical-by-descent (π^) value ≤ 0.1875;
- problematic inferred gender (command 'plink --sex-check');
- outliers according to heterozygosity (Budapest: 843×3426794, Manchester: 1083×3542437, Total: 1926×3513586);
- a minor allele frequency (MAF) of 0.01 reapplied (Budapest: 843×3424013, Manchester: 1083×3530244, Total: 1926×3504325).

Principal components were also calculated to control for genetic heterogeneity before the 'plink --sex-check' step:

- LD-pruning step (command 'plink --indep-pairwise 1500 150 0.2') to generate the first 10 principal components (PCs) for every individual.

Finally, SNPs were then filtered for the candidate loci reported by Gormley et al. and their 10,000 bp vicinity yielding 36579 candidate polymorphisms in 1815 individuals as a final dataset for subsequent analysis.
