# Supplementary material for: A replication study separates polymorphisms behind migraine with and without depression
Source: PLoS One. 2021 Dec 31;16(12):e0261477. doi: 10.1371/journal.pone.0261477 (PMC8719675; doi:10.1371/journal.pone.0261477)
Supplement: S2 Appendix — (PDF) [file pone.0261477.s021.pdf]

S2 Appendix Additional information on methods.

Bayesian relevance analysis

Bayesian relevance analysis [1] performs a random walk using a Markov Chain Monte Carlo (MCMC) method in the space of directed acyclic graphs (DAGs) [2, 3]. The transition between states of DAG space, i.e. between possible DAG structures, is facilitated by edge operators that delete, invert or insert a directed edge [4]. At each step the proposed transition is evaluated by a Bayesian Dirichlet structural score which incorporates a likelihood term that measures how likely the data set is given the current structure, and a prior term which assesses the probability of various DAG structures [5]. In short, a better score means that the structure fits the data better. If the proposed transition leads to a state with a better structure, then the transition is accepted. On the other hand, if the transition would lead to a state that corresponds to a structure with a lower score, then the transition is only allowed with a limited probability, which reflects the ratio of the scores of the proposed and the current structures. In an optimal case, the MCMC process reaches a stationary state after a number of steps. The result is a set of DAG structures (i.e. samples taken at each step) which then can be used to evaluate various structural properties such as direct edges or strong relevance by applying Bayesian model averaging [6]. Note, that samples taken during the burn-in period, which is approximately the interval preceding convergence, are discarded.

Strong relevance

The posterior probability of a direct edge between nodes X and Y, which can be interpreted as a direct relationship between variables X and Y, can be computed as the ratio of DAG structures (models) that contain the direct edge from variable X to Y and all sampled DAG structures (models) [2]. In addition, the strong relevance (referred to as relevance in the paper) of variable X with respect to Y means that there is either a direct edge between X and Y, or X is in a

structural interaction with Y (e.g. X→Z←Y) [7]. Therefore, the posterior probability of strong relevance is the sum of the posterior probability of direct edges (X→Y and X←Y) and that of interaction terms (X→Z←Y). In other words, the probability of strong relevance of X with respect to Y is the probability that X is in the minimal Markov blanket of Y [7].

For example, let X denote a SNP and Y denote migraine as the target variable, if the direct relationship X→Y exists in 20 out of 25 possible DAG structures then its posterior probability is 0.8. Furthermore, if Y→X does not appear in any of the structures and X is not involved in interactions, then the posterior probability of strong relevance of X with respect to Y is also 0.8. Otherwise, the sum of these probabilities (direct relationship and interaction) equals the probability of strong relevance.

References

1. Antal P, Millinghoffer A, Hullám G, Szalai C, Falus A. A Bayesian View of Challenges in Feature Selection: Feature Aggregation, Multiple Targets, Redundancy and Interaction. In: Yvan S, Huan L, Iñaki I, Louis W, Yves Van de P, editors. Proceedings of the Workshop on New Challenges for Feature Selection in Data Mining and Knowledge Discovery at ECML/PKDD 2008; Proceedings of Machine Learning Research: PMLR %J Proceedings of Machine Learning Research; 2008. p. 74--89.
2. Friedman N, Koller D. Being Bayesian about network structure: A Bayesian approach to structure discovery in Bayesian networks. Machine Learning. 2003;50(1-2):95-125. doi: 10.1023/A:1020249912095.
3. Madigan D, Andersson SA, Perlman MD, Volinsky CT. Bayesian model averaging and model selection for markov equivalence classes of acyclic digraphs. Communications in Statistics - Theory and Methods. 1996;25(11):2493-519. doi: 10.1080/03610929608831853.
4. Giudici P, Castelo R. Improving Markov Chain Monte Carlo Model Search for Data Mining. Machine Learning. 2003;50(1):127-58. doi: 10.1023/A:1020202028934.
5. Buntine W. Theory refinement on Bayesian networks. Proceedings of the Seventh conference on Uncertainty in Artificial Intelligence; Los Angeles, CA: Morgan Kaufmann Publishers Inc.; 1991. p. 52–60.
6. Hoeting JA, Madigan D, Raftery AE, Volinsky CT. Bayesian Model Averaging: A Tutorial. Statistical Science. 1999;14(4):382-401.
7. Pearl J. Causality: Models, reasoning, and inference. New York, NY, US: Cambridge University Press; 2000. xvi, 384-xvi, p.
